# General practitioners' views on reattribution for patients with medically unexplained symptoms: a questionnaire and qualitative study

**DOI:** 10.1186/1471-2296-9-46

**Published:** 2008-08-19

**Authors:** Christopher Dowrick, Linda Gask, John G Hughes, Huw Charles-Jones, Judith A Hogg, Sarah Peters, Peter Salmon, Anne R Rogers, Richard K Morriss

**Affiliations:** 1 Division of Primary Care, School of Population, Community and Behavioural Sciences, University of Liverpool, Liverpool L69 3GB, UK; 2 National Primary Care Research and Development Centre, 5th Floor Williamson Building, University of Manchester, Oxford Road, Manchester M13 9PL, UK; 3 The Lache Health Centre, Hawthorn Road, Chester, CH4 8HX, UK; 4 Division of Psychiatry, School of Population, Community and Behavioural Sciences, University of Liverpool, Liverpool L69 3GB, UK; 5 Division of Psychology, School of Psychological Sciences, University of Manchester, Manchester M13 9PL, UK; 6 Division of Clinical Psychology, School of Population, Community and Behavioural Sciences, University of Liverpool, Liverpool L69 3GB, UK; 7 Division of Psychiatry, School of Community Health Sciences, University of Nottingham, South Block, A Floor, Queen's Medical School, Nottingham, NG7 2UH, UK

## Abstract

**Background:**

The successful introduction of new methods for managing medically unexplained symptoms in primary care is dependent to a large degree on the attitudes, experiences and expectations of practitioners. As part of an exploratory randomised controlled trial of reattribution training, we sought the views of participating practitioners on patients with medically unexplained symptoms, and on the value of and barriers to the implementation of reattribution in practice.

**Methods:**

A nested attitudinal survey and qualitative study in sixteen primary care teams in north-west England. All practitioners participating in the trial (n = 74) were invited to complete a structured survey. Semi-structured interviews were undertaken with a purposive sub-sample of survey respondents, using a structured topic guide. Interview transcripts were used to identify key issues, concepts and themes, which were grouped to construct a conceptual framework: this framework was applied systematically to the data.

**Results:**

Seventy (95%) of study participants responded to the survey. Survey respondents often found it stressful to work with patients with medically unexplained symptoms, though those who had received reattribution training were more optimistic about their ability to help them. Interview participants trained in reattribution (n = 12) reported that reattribution increased their confidence to practice in a difficult area, with heightened awareness, altered perceptions of these patients, improved opportunities for team-building and transferable skills. However general practitioners also reported potential barriers to the implementation of reattribution in routine clinical practice, at the level of the patient, the doctor, the consultation, diagnosis and the healthcare context.

**Conclusion:**

Reattribution training increases practitioners' sense of competence in managing patients with medically unexplained symptoms. However, barriers to its implementation are considerable, and frequently lie outside the control of a group of practitioners generally sympathetic to patients with medically unexplained symptoms and the purpose of reattribution. These findings add further to the evidence of the difficulty of implementing reattribution in routine general practice.

## Background

Approximately 20% of patients present physical symptoms in primary care which general practitioners (GPs) are unable to explain by physical disease [1,2]. These patients frequently receive extensive investigation, referral and treatment for medically unexplained symptoms (MUS). However such interventions are often ineffective [3-6] and these clinical encounters can lead to dissatisfaction on the part of both doctors and patients [7]. For patients it may create a frustrating dependence on primary care consultations which generates ambivalence, alienation and unhappiness with medical contact [8].

**Figure 1:**
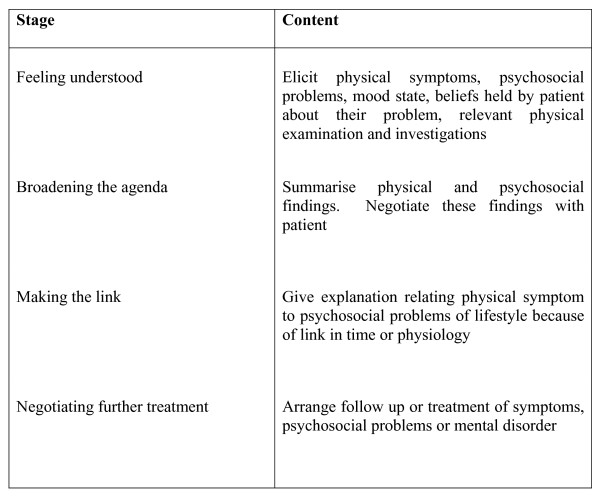
Content of the Reattribution Intervention.

Reattribution is a structured intervention, designed to provide a simple explanation of the mechanism of a patient's MUS, through negotiation and other features of patient-centred communication, and to be delivered during routine consultations [9]. It has four stages: enabling the patient to feel understood; broadening the agenda beyond physical symptoms; making the link with psychosocial issues; and negotiating further treatment [10] (See Figure 1).

Early studies suggested reattribution had promise as a simple and effective intervention that GPs could employ on a routine basis for patients with MUS [11-14]. However the results of recent randomised trials indicate that, whilst the techniques of reattribution can successfully be taught to GPs, and do have a measurable impact on practitioner behaviour in consultations about MUS, it is more difficult to identify tangible or lasting benefits in terms of improved outcomes for patients [15-17].

The reasons for the apparently limited efficacy of reattribution in routine clinical practice are complex. They include: the impact of contextual factors within the organisation and delivery of primary care [9]; and the expectations of patients with MUS about the type of care they should receive from GPs. Patients may have concerns that their needs for treatment, explanation and support will not adequately be met by doctors who appear to be looking for psychosocial rather than medical explanations for their symptoms [18].

The attitudes, experiences and expectations of GPs themselves are also important, since they are essential to the successful implementation of any new method for managing MUS in primary care. It is therefore necessary to ascertain what GPs value about reattribution as an intervention for MUS, and the feasibility of implementing it in everyday clinical practice. In order to explore these issues, we conducted a questionnaire survey and undertook qualitative interviews with GPs who were taking part in an exploratory RCT of reattribution training in north-west England [10].

## Methods

The setting for this study was 16 practices in the north-west of England. The study sample was composed of the practitioners (73 GPs and one nurse prescriber (NP)) taking part in an exploratory randomised control trial of the effects of reattribution training on GP communication behaviour with patients presenting with persistent MUS. The six hour (two session) training programme was delivered by a health facilitator to all members of the practice team [10]. The study was approved by the North West multi-centre research ethics committee.

Practices had a median of four (range two to 10) GPs. Three practices served inner city populations, one a rural population and 12 practices urban populations that included some inner city areas. The GPs were mostly aged 35 to 50 (n = 45, 60%), with 10 (13%) under the age of 35 years and 19 (25%) over the age of 50 years. Thirty-eight (51%) were male. There were no differences between the training groups in terms of GP or practice characteristics.

All 74 practitioners were invited to participate in an attitudinal survey. Practitioners who had received reattribution training (RT) were asked for their views on: patients with persistent MUS (three statements); managing patients with MUS (four statements); the process of RT (five statements); and putting RT into practice (six statements). Practitioners who had not received RT were given only the first two sets of statements. Ten statements were framed negatively, and seven positively. Responses were in the form of a 5 point Likert scale. Responses were analysed using SPSS version 15.0, first generically and then to identify significant differences between practitioners who had or had not received RT.

Qualitative interviewees were purposively selected by MT and JGH (with advice from JAH, SP, LG and CD) from questionnaire respondents, in order to generate as wide a variation of views as possible. The sampling criteria included: respondents from each practice (to ensure all participating practices were represented); the practice setting (rural, suburban; inner city); the number of patients recruited by GPs into the trial; entry into training or control arm of the trial; gender and age of the GP; and participants' responses to two attitudinal questions about patients with MUS: '*I think patients with MUS take up too much time, which I could use more productively with other patients'*; and '*There's a lot of patients for whom reattribution does not work'*.

Twenty four interviews were conducted, by MT and JGH, between August 2005 and May 2006, up to 31 months after practitioners were recruited into the study. Sixteen interviewees were women. Three were aged under 35, 14 were between 35 and 50, and seven were over 50. Nine worked in inner city practices, 14 in suburban practices, and one was from a rural practice. They had recruited between 0 and 11 patients to the trial (mean 3.3; median 3). Twelve were in the training arm of the trial and 12 were in the control arm. The selected sample represented the full range of responses to the two identified survey questions.

A topic guide invited participants' views on patients with persistent MUS and their management of MUS. For those who had been trained in reattribution, further prompts invited views on the value and benefits of reattribution, and also on any barriers to its implementation in practice. Interviews were held in person, in the respondents' place of work at a time of their choosing. Respondents were remunerated for taking part.

Interviews were audio-taped and transcribed verbatim. Transcripts were read and re-read by JGH, CD and SP to familiarize and immerse the researchers in the data. Patterns and themes within the transcripts were identified and notes made within the margins of transcripts. Following principles of grounded theory research, meetings were held with the whole research team to discuss and identify key issues, concepts and themes arising from the data, and to group them thematically to construct a conceptual framework [19]. The thematic framework was applied systematically to the data. When devising the conceptual framework the research team were mindful of the aims of the research. All transcripts were indexed by JGH and then collated together within each code. The range and dimensions of each category and sub-category were identified. Conceptual connectors were sought to understand how categories and subcategories were linked at the level of properties and dimensions.

## Results

### Attitudinal Survey

Seventy (95%) of the 74 practitioners in the sample responded to the survey. Of these, half had received RT. The attitudinal statements, and range of responses, are presented in Table 1.

**Table 1:** Attitudinal survey of practitioners participating in MUST

Statement	Agree completely	Agree partly	Unsure	Disagree partly	Disagree completely	missing
about patients with PMUS* N = 70	N (%)	N (%)	N (%)	N (%)	N (%)	
I enjoy consultations with patients who have PMUS	3 (4)	17 (24)	14 (20)	30 (43)	6 (9)	-
I think patients with PMUS take up too much of my time, which I could use more productively with other patients	4 (6)	15 (21)	5 (7)	31 (44)	15 (21)	-
I find that patients with PMUS often cause me considerable stress	8 (11)	43 (61)	3 (4)	11(16)	5 (7)	-
I don't think it's worth trying to do much with patients who have PMUS	0 (0)	3 (4)	5 (7)	28 (40)	33 (47)	1 (1)
I find that patients with PMUS present me with interesting diagnostic challenges	13 (19)	38 (54)	5 (7)	12 (17)	1 (1)	-
I find that patients with PMUS present me with interesting therapeutic challenges	18 (26)	40 (57)	3 (4)	7 (10)	1 (1)	1 (1)
I often don't know how to help patients who have PMUS	8 (11)	33 (47)	3 (4)	21 (30)	4 (6)	1 (1)
						
about reattribution training N = 35						
The reattribution training programme did not really teach me anything new	2 (6)	11 (31)	4 (11)	12 (34)	6 (17)	-
It was easy to find time to concentrate on the training programme, despite the pressure of clinical work in my practice	4 (11)	10 (29)	1(3)	14 (40)	6 (17)	-
The training programme would have been better if it included more formal lectures	0 (0)	2 (6)	6 (17)	8 (23)	19 (54)	-
I would have been more inclined to engage with the training programme if I'd been paid to attend the sessions	3 (9)	5 (14)	6 (17)	11 (31)	10 (29)	-
In general, I enjoyed the training programme	15 (43)	15 (43)	3 (9)	2 (6)	0 (0)	-
I have found it easy to put reattribution into practice	6 (17)	20 (57)	3 (6)	6 (17)	0 (0)	-
I have already forgotten some of the reattribution stages	4 (11)	20 (57)	1 (3)	5 (14)	5 (14)	-
I often need several consultations with patients to achieve all the reattribution stages	18 (51)	14 (40)	1 (3)	2 (6)	0 (0)	-
There are lots of patients with whom reattribution does not work	5 (14)	12 (34)	13 (37)	4 (11)	1 (3)	-
In general, putting reattribution into practice makes my consultations with these patients more enjoyable	7 (20)	13 (37)	9 (26)	6 (17)	0 (0)	-
In general, putting reattribution into practice makes my consultations with these patients quicker	1 (3)	6 (17)	10 (29)	13 (37)	5 (14)	-

Respondents tended not to enjoy consultations with patients with persistent MUS. Although most did not think these patients took up too much of their time, a substantial majority reported that they often caused them considerable stress. They thought these patients are worth trying to help, and that they present interesting diagnostic and therapeutic challenges. However, most respondents reported that they often do not know how to help these patients.

There were two significant differences in responses between practitioners who had received RT and those who had not, when analysed using one way ANOVA. Practitioners who had received RT were more likely to report that patients with MUS take up too much time (mean scores 3.26 vs. 3.83, F = 4.062, p = 0.048). However they were less likely to report that they often did not know how to help these patients (mean scores 3.11 vs. 2.29, F = 9.188, p = 0.003).

Amongst respondents who had received RT, most reported that they found the training enjoyable, and disagreed that it had not taught them anything new. However most thought it was not easy to find time to concentrate on RT. Few thought that lectures or payment would have improved their experience of or engagement with RT.

Trained respondents reported that putting RT into practice was fairly easy, and that it generally made consultations with MUS patients more enjoyable, although not quicker. However, most agreed that they had already forgotten some of the reattribution stages. Almost all considered that it often needed several consultations to complete all stages of reattribution, and very few disagreed with the statement that there are lots of patients for whom reattribution does not work.

### Qualitative Interviews

In this section of the paper we focus on the responses of twelve GPs who had received training in reattribution to questions about: a) limitations and benefits of reattribution; and b) barriers to its implementation in routine clinical practice.

#### Limitations and benefits of reattribution

**Figure 2:**
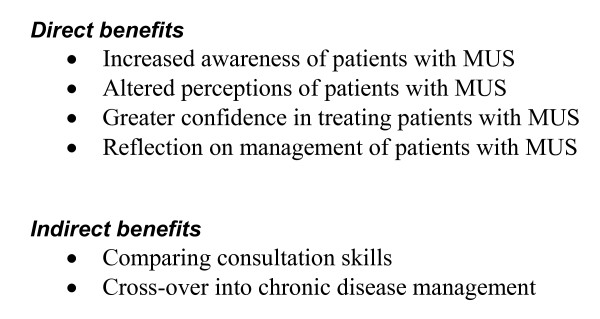
GPs views of benefits of reattribution training.

Participants voiced some criticisms of the training in reattribution. Some thought they were already doing it: '*it didn't give me anything new*' [PGP11]. Concerns were also expressed about the time commitment, aspects of the format and content of the training programme and – commonly – the lack of reinforcement: '*supervision would have been nice*' [PGP12]. However, they described both direct and indirect benefits from learning about reattribution (see Figure 2).

Many indicated that training in reattribution increased their awareness and altered their perception of patients with MUS:

*It er, helped one to sort of spot the situations perhaps a bit, bit better, increased one's awareness, and you know, increased perhaps just really the, the trying to explain to patients the, the way the symptoms developed if you like from, well often from anxiety or other causes* [PGP23]

*altered my perception a bit, its easy to get stale and view that group of patients as difficult or troublesome or irksome at times because we're not always at our best every time* [PGP11]

Some found that reattribution training had a positive impact on their consultations, increasing their confidence in discussing MUS, and enabling them to reflect on their management decisions:

*I'd like to think that I do go a little bit more into other agendas, other issues that might be fuelling the symptoms that they've got and try and approach those other problems rather than just focusing on a prescription for something for pain* [PGP13]

*It's made me stop and think why I am referring this person yet again. I think there are not an awful lot of examples, since we've been doing the training come to mind referring here there and everywhere, but just in general because of the training I've thought lets just stop and look, what are we actually achieving* [PGP02]

Some GPs valued the additional structure provided by reattribution:

*I think it did, it did formalise it but I think it wasn't something that I would have been embarrassed about or found it difficult to do to discuss the physiology of anxiety and how it might produce physical symptoms* [PGP09]

Respondents also reported indirect benefits from reattribution training, which were unrelated to their management of MUS. As well as '*recharging the batteries*' (PGP11) and doing something new '*to put on the CV*' (PGP18), the training programme was seen as a valuable opportunity for GPs to compare consultation skills with colleagues within their own practice. Some respondents had also used reattribution in their consultations with non-MUS patients:

*The emphasis it puts on explanation and so on, I think that, you can carry across into other areas....I think some of the chronic diseases most probably been some cross-over* [PGP23]

#### Barriers to implementing reattribution

**Figure 3:**
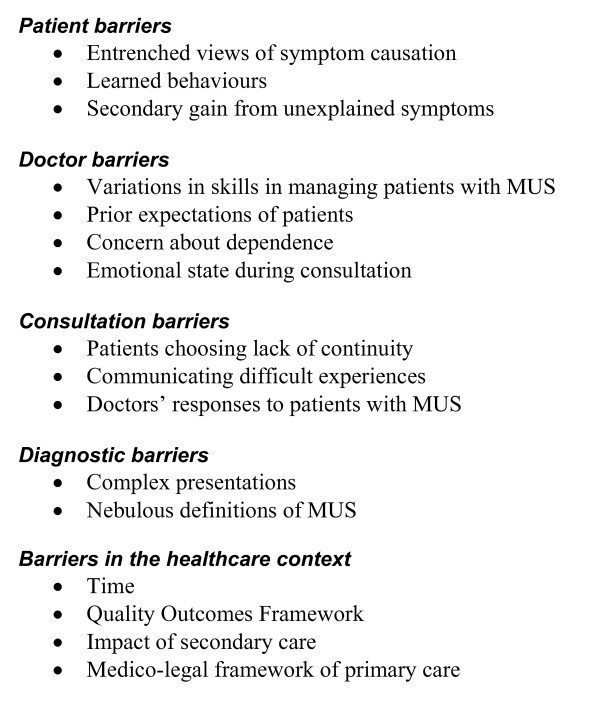
GPs' views of barriers to implementation of reattribution for patients with MUS.

Respondents described many barriers to implementing reattribution in routine general practice. We categorise these as arising from the patient, the doctor, the consultation, the diagnosis, and the context of care (see Figure 3).

##### Patient barriers

The attitudes of patients with MUS were seen as important barriers by many GPs. They frequently described how patients may have entrenched views that their symptoms have an organic basis:

*Well it's their mindset isn't it? It's their belief that there is a physical cause* [PGP18]

Respondents expressed various and occasionally contradictory opinions about the impact of gender, intelligence, class and ethnicity on the likelihood of patients' holding fixed beliefs in physical causation of MUS. However they all considered the presence of such beliefs to be a fundamental barrier to successful reattribution.

Many GPs considered that patients' belief in organic causes of MUS could be perpetuated by family members since childhood, becoming a learnt behaviour or a strategy for coping with unhappiness.

*These are patients who've learnt to present their unhappiness in physical ways and they may have been in a family where they went to the doctor with every little bit of pain so their mothers might have been frequent attenders and its been a sort of learnt behaviour....I think they must have learnt that some behaviour is advantageous* [PGP01]

Respondents thought patients could derive benefit from their symptoms, in relation to work, state benefits or family support:

*The patient might have gains from their, from their symptoms as well. So they might have, there might be gains about getting benefits or support from the university or support from key people in their lives* [PGP22]

##### Doctor barriers

Respondents were aware of variations in communication skills and the ability to deliver the stages of reattribution in routine clinical settings:

*It's like basic training. Sort of communication skills, history taking.....some people are better than others* [PGP18]

They were aware that their prior expectations of the patient could influence how likely they were to attempt reattribution.

*With certain people, before they've come in, you've already perceived it's going to be difficult because that's your prior expectation, your prior knowledge of them.....I mean and they could be coming with something totally different, but your initial reaction is "oh my god"* [PGP16]

GPs expressed concern that getting involved with reattribution may lead to the patient becoming dependent on them:

*Not that the relationship has broken down but you don't want to have such a close relationship with them that they're relying on you* [PGP09]

Two respondents acknowledged that their own emotional state or mood affected the likelihood of using reattribution.

*[it depends] how you're feeling that day* [PGP04]

*I try [to reattribute] on a good day. On a bad day I just try and give the least damaging medication and do the least number of referrals* [PGP08]

##### Consultation barriers

Respondents considered that patients' consulting behaviour can make it difficult to apply reattribution, particularly if they choose to consult with different GPs within a practice.

*If they're at the more difficult end of the spectrum they will often deliberately pick off a locum doctor or a registrar to get what they want, they have an agenda* [PGP11]

However, GPs were not always critical of their patients' unwillingness or inability to communicate with their doctor sufficiently to allow them to broaden the agenda.

*[There are] occasions where you do have a very good relationship but something is too, either too painful or too private to share with you* [PGP16]

*Its difficult because sometimes you can see..., I've got one patient that all her symptoms started after the break up of her marriage and she can't see the link, and because of the type of person she is, she's extremely proud and you don't want to keep banging on about it* [PGP04]

Some GPs acknowledged that their own responses to patients during consultations could impede reattribution:

*I find some patients quite easy to get a rapport with, and it's the ones that you really have little in common with, or not even, you can get on with people that you have nothing in common with but when there's just something that brushes you up the wrong way and I find that quite hard to turn around* [PGP04]

##### Diagnostic barriers

Many GPs commented that patients do not present solely with MUS, but rather have numerous symptoms, some with an organic basis and some with a psychological underpinning. The presence of organic and medically unexplained symptoms can make it harder for GPs and patients to disentangle the causes of their symptoms.

*Most patients I find have medically unexplained and medically explainable symptoms, its very complicated and in my experience its very rare to have somebody just coming in with unexplained symptoms because nowadays if we're diagnosing more people with hypertension than diabetes and whatever, so we are giving people labels for certain conditions anyway* [PGP09]

The difficulty of labelling patients as 'definitely' having MUS was also seen as a potential barrier in deciding who and when to use reattribution:

*This is a bit more nebulous, you've got to think more deeply about it and it's not as though the patient is coming in and you catch the MUS and label the MUS and that maybe why its higgilty piggilty cos I can't label it and say right that's your MUS and this is what we are going to do. And that makes it more difficult, you know whom we will we start to reattribute, whom wants to get to think, to think what causes their symptoms and how early we do it* [PGP02]

##### Barriers in the healthcare context

Respondents commonly reported insufficient time to deal effectively with MUS patients, with busy surgeries and brief appointments pushing them away from reattribution and towards short-term solutions.

*Time pressure is such that you're looking at certain quick fixes, you may not be consciously looking outside the box* [PGP02]

Some GPs thought that the Quality Outcomes Framework (QOF) compounded these time pressures, and detracted from their ability to reattribute.

*It's probably impacted on every consultation in the fact that you need to collect data. So often it's difficult to spend the time pursuing things that you might have pursued before, because actually you've got to record their height, weight, body mass, or whether they smoke and all practical stuff, and that detracts from being able to pick up problems. ...So I think it does actually alter consultations in those situations* [PGP22]

There was a common view that contact with secondary care was unhelpful, with an increase in inappropriate physical diagnoses, and greater entrenchment in symptoms.

*It does tend to be that if, if they are often in secondary care that they are given a physical diagnosis whether there is one or not....That can support their belief in a physical problem* [PGP21]

Some respondents saw it as their role to protect patients from this potential source of harm:

*You see these people getting referred to the hospital with back pain and the next thing you know some bright spark is going to operate on them and you think 'What!' ....Maybe we're here in a way as a gateway to try and prevent harm as well as anything else* [PGP06]

However others were aware of the potentially punitive medico-legal framework within which they operate, which pushes them towards an over-emphasis on the identification or exclusion of physical illness

*You're never criticised for over-diagnosing and inappropriately over-treating patients but you can lose your job for missing a diagnosis, so the whole thing tips completely the wrong way, and not in the patients favour in that sense* [PGP08]

## Discussion

### Summary of findings

Responses to the questionnaire survey indicated that these practitioners were generally sympathetic to patients with MUS, but often found them a source of stress and difficult to help. Although respondents who had received RT tended to be less positive in their views about patients with MUS, they were more confident that they knew how to help them. Respondents generally enjoyed the process of training, but often forgot key elements of reattribution, and presented mixed views about the ease and practicality of its implementation in practice.

The participants in the qualitative study who had received RT described direct benefits from reattribution, including a greater sense of confidence and coherence when consulting with patients with MUS, and also indirect benefits including sharing consultation skills and application of new skills in the management of chronic disease. However they also reported multiple interlinking barriers to the successful implementation of reattribution in routine practice. Barriers at the patient level included the perception of entrenched views about physical causes of symptoms that were not amenable to change, compounded by learned behaviours and secondary gains. Doctor barriers included lack of skill, negative expectations of the patient, concern about encouraging dependence and personal emotional states. Barriers within the consultation included patients choosing not to consult with a regular doctor or being unwilling to share private information, and GPs' responses to particular patients. Diagnostic barriers were the frequent combination of explained and unexplained symptoms, and the nebulous definitions of MUS. Barriers in the healthcare context included time, organisational requirements, concerns about the negative impact of secondary care and the fear of the medico-legal consequences of missing physical diagnoses. Additionally, they report the complexity of working with degrees of uncertainty which are difficult to resolve.

### Strengths and limitations

We have previously demonstrated that this group of GPs is more sympathetic towards patients presenting with MUS, and places greater value on their own psychosocial skills in relation to such patients, than GPs who do not wish to take part in research of this kind [20]. Furthermore, we have shown in this study that they see substantial direct and indirect benefits of reattribution. They may therefore be taken as a group of doctors who tend to be well disposed towards the implementation of reattribution within routine clinical practice in primary care. For these reasons, the many and various concerns they express about the limited scope for implementing reattribution deserve serious consideration – coming from a group of 'critical friends' rather than from neutral or even hostile observers. We see this as a major strength of this study.

By definition, therefore, a limitation of this study is that the views of these GPs cannot be assumed to be representative of their discipline: the relatively high proportion of female participants in the qualitative survey, compared to the proportion of female GPs in England as a whole also indicates unrepresentativeness. Secondly, while most of the barriers they discuss are common across all healthcare settings, there are several organisational constraints that are specific to healthcare in the UK, such as ten minute consultations and the Quality and Outcomes Framework [21], and may not be generalisable elsewhere. We also acknowledge that the metaphor of 'barriers' used by the research team is based on the assumed ability of participants to implement change, while operating within a highly complex set of diagnostic and organisational contexts [22].

### Comparisons with existing literature

This is the first study to describe the views of GPs about the benefits and barriers to implementing reattribution in routine clinical practice. Our methods, combining structured attitudinal responses with semi-structured interviews with a purposive sample of GPs, were chosen to allow as wide a range of perspectives as possible to emerge. This paper is therefore takes forward previous research in this field, which has demonstrated changes in GPs' attitudes to patients with MUS following reattribution by quantitative means [11,15,23], but has not combined this with qualitative methods to gather their detailed views about reattribution and how it may work in practice. In apparent contrast to our quantitative finding, Danish GPs who had been trained in reattribution reported less concern about time spent with MUS patients [23]: however this study reported within-subject differences over time, whereas we compared attitudes of trained and non-trained respondents.

These GPs described a complex and interlinking set of barriers to the implementation of reattribution. Some of these observations could be regarded as stereotypical and partial: the frequent tendency to blame time constraints for difficulties in applying reattribution in practice can be seen as an example of a 'culturally honourable' excuse, a means of mitigating responsibilities when behaviour is questioned [24].

Most of the barriers described by these GPs, however, indicate a more nuanced and reflective response to the problems of managing patients with MUS, including a ready acknowledgement not only of the difficult diagnostic and organisational context within which they have to operate, but also of the significance of their own attitudes and personal responses. Although they value the use of reattribution for patients with MUS, they do not always feel able to take on the work involved, or to shoulder the burden of responsibility that may ensue [25]. In this sense at least, the barriers they describe may not merely be obstacles to success, but may sometimes have a protective function for doctors acting within a difficult arena.

There are important differences and synergies between GP and patient perspectives. GPs considered patients with MUS to have fixed physical attributions. However this view is not supported by recent evidence from primary care [26]. Rather, patients choose to present physical attributions to their GP whilst withholding psychological components of their illness beliefs [27], which is a further barrier to GPs attempts to reattribute. It appears that both GPs and patients consider the problem to be complex, while believing that the other party holds a more simplistic view than their own. Despite recognising the value in preventing further contact with secondary resources, GPs admitted reluctance in abandoning pathology investigations. A driving force for this, from GPs' perspectives, is the medico-legal framework within which they work. Elsewhere patients describe pursuing a similar agenda; also out of fear of missing disease, either now or if presenting with future problems [27].

## Conclusion

It is important to recognise that both patients with MUS, and the GPs they encounter are heterogeneous groups, with competing attributions on one side [28], and varying degrees of empathy and skill on the other. A key question, therefore, is how to establish a common understanding of illness and treatment expectations. Our findings indicate the need for greater specificity, with regard to the patients and circumstances in which the techniques of reattribution may successfully be applied. This is of relevance both to future research and to current practice.

Some of the barriers to reattribution reported by GP respondents were seen as immutable, such as the coexistence of unexplained and explained symptoms and (perhaps) the fixed beliefs of patients. It may therefore be useful for educators to focus on addressing those barriers to psycho-social interventions which appear more amenable to change, such as the need for increased skill, or for different medical attitudes towards patients with MUS. A stepped care approach, assuming only a basic level of knowledge, interest and skill amongst the majority of GPs, may also address the diagnostic and organisational barriers reported by our respondents [29].

One way forward would be to take the ideal type of circumstances for the delivery of reattribution as described by these practitioners: patients whose MUS are associated with fluid causative beliefs, have no family expectations of illness and see nothing to gain from their symptoms; in consultations with regular GPs who feel well disposed towards their patients and comfortable in themselves, are confident in their consultation skills, can tolerate diagnostic uncertainty, and feel concerned neither about time pressures nor punitive organisational or medico-legal constraints. These appear to be the circumstances in which reattribution is most likely to prove successful. It may therefore be wise to demonstrate efficacy in relatively calm conditions first, before braving the elements and exploring more troubled waters.

## Competing interests

The authors declare that they have no competing interests.

## Authors' contributions

CD designed and analysed the questionnaire and drafted the manuscript. JAH organised and collated the attitudinal survey. JGH undertook qualitative interviews and initiated the qualitative analysis. CD, JGH and SP completed the qualitative analysis. RKM, LG, CD, PS, SP and ARR conceived the study, and participated in its design and coordination. All authors read and approved the final manuscript.

## Funding

Medical Research Council (Grant reference number G0100809), Mersey Care NHS Trust and Mersey Primary Care R&D Consortium.

*Ethical approval*: This study was approved by the North West multi-centre research ethics committee.

## Pre-publication history

The pre-publication history for this paper can be accessed here:


